# Aiming for the top: non-cell autonomous control of shoot stem cells in Arabidopsis

**DOI:** 10.1007/s10265-020-01174-3

**Published:** 2020-03-07

**Authors:** Michael Fuchs, Jan U. Lohmann

**Affiliations:** Department of Stem Cell Biology, Centre for Organismal Studies (COS), Heidelberg University, Im Neuenheimer Feld 230, 69120 Heidelberg, Germany

**Keywords:** Plant development, Stem cells, Shoot apical meristem, WUSCHEL, Protein mobility

## Abstract

In multicellular organisms, not all cells are created equal. Instead, organismal complexity is achieved by specialisation and division of labour between distinct cell types. Therefore, the organism depends on the presence, correct proportion and function of all cell types. It follows that early development is geared towards setting up the basic body plan and to specify cell lineages. Since plants employ a post-embryonic mode of development, the continuous growth and addition of new organs require a source of new cells, as well as a strict regulation of cellular composition throughout the entire life-cycle. To meet these demands, evolution has brought about complex regulatory systems to maintain and control continuously active stem cell systems. Here, we review recent work on the mechanisms of non cell-autonomous control of shoot stem cells in the model plant *Arabidopsis thaliana* with a strong focus on the cell-to-cell mobility and function of the WUSCHEL homeodomain transcription factor.

## Stem cells are maintained in stem cell niches

Stem cells are cells that are undifferentiated, but retain the ability to divide and produce new cells. Daughter cells either remain stem cells or differentiate into more specialised cells. Ongoing differentiation, however, restricts cells in their ability to proliferate and generate different cell types, while fully differentiated cells have lost the ability to divide. Therefore, stem cells are required, not only for development, but also for continuous tissue homeostasis to replenish expended cells. As such, they need to be tightly regulated: Uncontrolled over-proliferation of stem cells, for instance in disease, can be just as fatal as loss of stem cell activity, since in both cases cellular composition is disturbed and tissue function can no longer be sustained.

Stem cells are not only a prerequisite for complex multicellularity, but like multicellularity itself have developed independently in the plant and animal kingdom. While there are obviously differences between plant and animal (stem) cells, some underlying principles show surprising similarities, as similar challenges have been met with similar solutions. One example is the concept of the stem cell niche, a microenvironment wherein stem cells are initiated and maintained (Heidstra and Sabatini 2014). Once cells leave the stem cell niche, they gradually loose stem cell identity and progressively differentiate. The niche provides maintenance signals to control cell division, prevent differentiation and regulate niche exit. These signals can be mediated by direct interaction with niche elements, such as niche cells or the extracellular matrix, by biophysical cues or by diffusible signalling factors.

## The shoot apical meristem of *Arabidopsis thaliana*

**Fig. 1:**
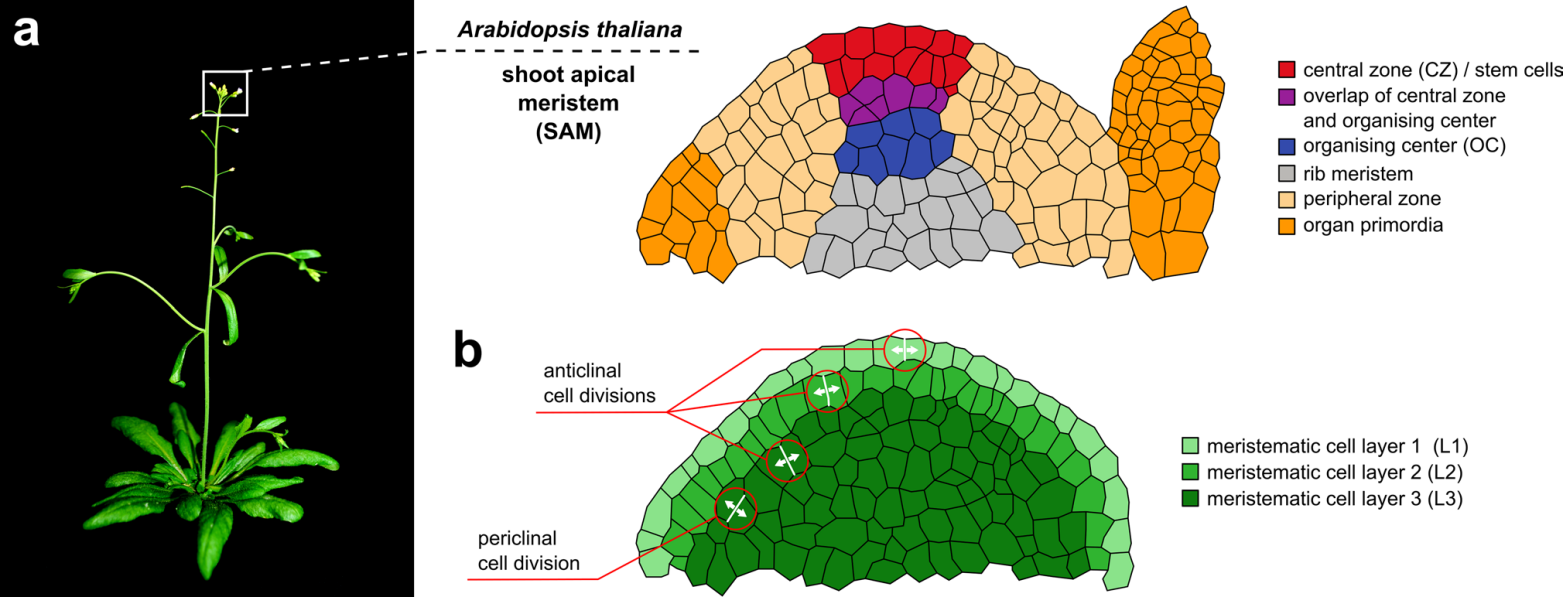
The shoot apical meristem of *Arabidopsis thaliana. ***a** Schematic representation of the shoot apical meristem (SAM) at the tip of the Arabidopsis shoot and of functional domains within the SAM. **b** Schematic representation of clonally distinct cell layers in the SAM. L1 and L2 originate from anticlinal cell divisions while cells in the L3 arise from anticlinal and periclinal divisions

**Fig. 2:**
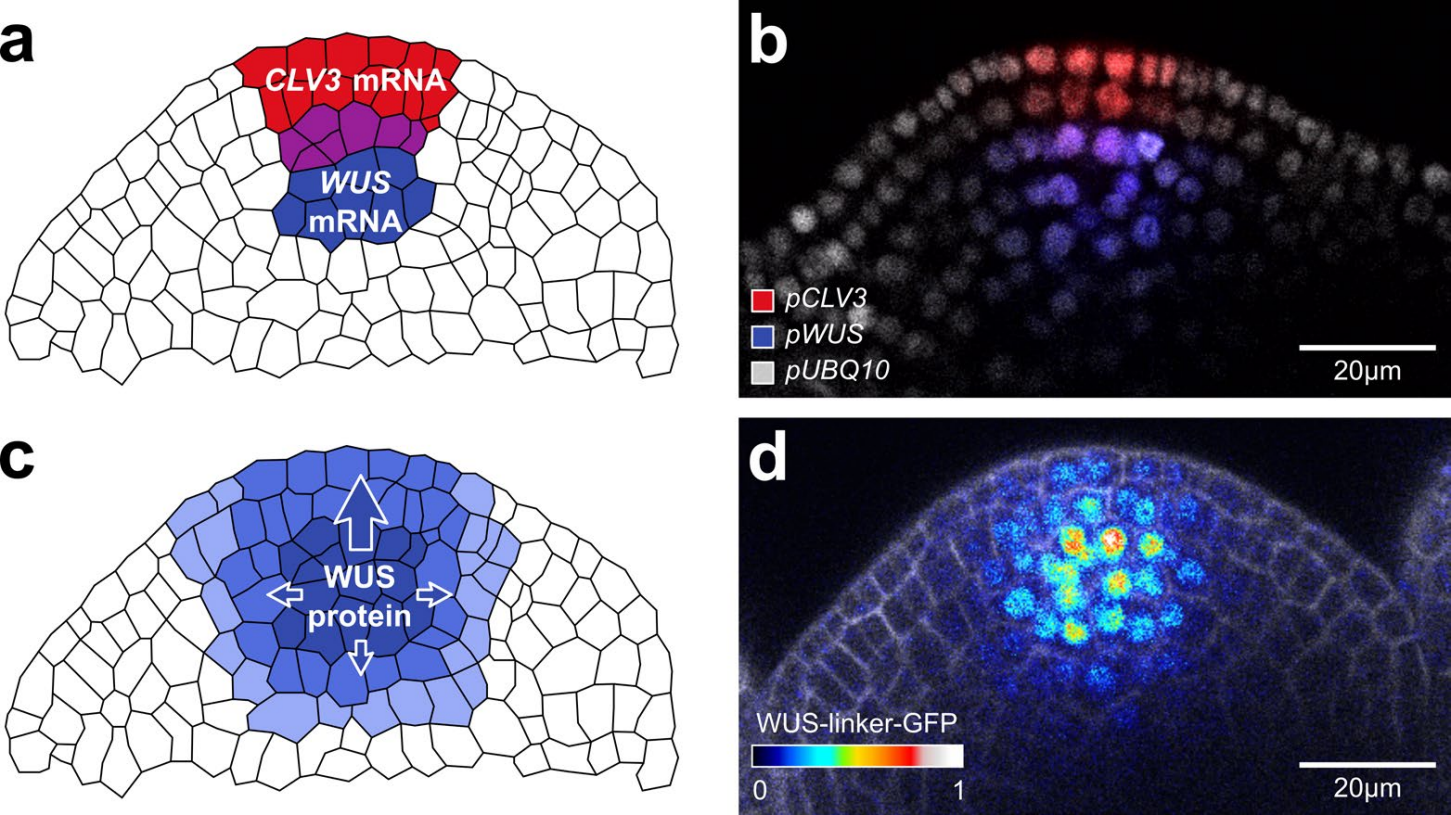
Localisation of key stem cell regulators in the SAM. **a** Schematic representation of the *CLV3* (red) and *WUS* (blue) mRNA expression domains. Note the overlap in the L3 (purple). **b** Confocal slice through the center of a *pCLV3* (red), *pWUS* (blue), *pUBQ10* (gray) triple reporter SAM. **c** Schematic representation of WUS protein localisation (intensity coded in blue). **d** Confocal slice through the center of a *pWUS::WUS-linker-GFP* rescue SAM. GFP was colour coded on a linear scale

An important stem cell niche in plants is the shoot apical meristem (SAM). In *Arabidopsis thaliana* (L.) Heynh., it is positioned at the shoot tip at the very top of the plant (Fig. 1a) and harbours a pool of pluripotent stem cells, which directly or indirectly is the source of all aerial tissue as well as the origin of the plant’s gametes. The SAM is a dome shaped structure with several molecularly distinct functional subdomains: The two uppermost cell layers, L1 and L2, are clonally distinct monolayers. Cells in these layers divide only anticlinally, that means perpendicular to the outer surface. In deeper cell layers, that is the third cell layer and all layers below, called L3, cells divide anticlinally, but also periclinally, that means parallel to the outer surface (Fig. 1b). All cell layers, L1, L2 and L3, maintain an independent pool of stem cells (Satina et al. 1940; Steward and Burk 1970). These stem cells are located in a domain in the centre of each layer, consequently named the central zone (CZ) (Fig. 1a) and are marked by the expression of the *CLAVATA3* gene (*CLV3*) (Fig. 2a, b) (Fletcher et al. 1999). When stem cells divide in the central zone, the surrounding cells are displaced and passively pushed laterally towards the peripheral zone or basally towards the rib meristem (Fig. 1a). Cells in the periphery divide more rapidly and in response to potent signals including the plant hormone auxin start differentiating as they are pushed further away from the CZ (Meyerowitz 1997; Reddy et al. 2004; Steeves and Sussex 1989). Once cells reach the boundary of the meristem, they are incorporated into organ primordia (Fig. 1a), which subsequently mature into leaves or flowers. Below the central zone, with slight overlap to the CZ, we find another distinct domain, the organizing centre (OC) (Fig. 1a), defined by the expression of the stem cell regulator *WUSCHEL* (*WUS*) (Fig. 2a, b), which will be discussed in detail later.

## The plant cell wall requires unique solutions for intercellular communication

Plant cells are encased in a stiff extracellular matrix, the cell wall. It spatially separates neighbouring cells by creating a diffusion barrier for large molecules or proteins and prevents migration of individual cells. With regards to the stem cell niche, this has two major consequences: First, while the relative position of a plant cell with respect to neighbouring cells may remain quite stable, its absolute position will not, due to cell division and cell elongation. For instance, a cell can start out being an L3 stem cell, but then be pushed out of the CZ into the OC where it needs to fulfil the function of an organising niche cell. Even later, further cell divisions of adjacent cells may move it out of the OC again to fully differentiate and execute yet another different organ function. This implies, that the molecular and functional domains of the SAM are position specific properties of the tissue as a whole, but are not determined by cell lineage. A second consequence of the cell wall encasing each plant cell is that direct cell-cell contact, even between neighbouring cells, is not possible. In addition, the cell wall limits free diffusion: While smaller factors, like for instance ions, phytohormones or small peptides such as the CLV3 peptide, may pass through the cell wall, this is not possible for larger molecules such as proteins or long RNAs, severely limiting the options for cell-cell communication and stem-cell-to-niche signalling. However, this limitation is mitigated by the presence of plasmodesmata. Plasmodesmata are cellular connections between neighbouring cells that are unique to plants. They are made from strands of cytoplasm, the cytoplasmic sleeve, that cross the cell wall and can include additional strands of endoplasmic reticulum (ER), called desmotubule. Transfer of cellular content is possible via the cytoplasm, via the ER lumen or via insertion into the plasma membrane or ER membrane, and is heavily regulated during development and by innate immunity responses. Plasmodesmata are narrow channels and it has been suggested that modulation of plasmodesmata size can be a means for trafficking regulation by limiting the size of molecules that can pass through, the so-called size-exclusion limit. A reduction of cell-cell connectivity via restricting plasmodesmata trafficking has been shown to be a hallmark of differentiation in developmental processes (Crawford and Zambryski 2001; Zambryski 2004). In innate immunity, complete closure of plasmodesmata is a mechanism to seal off cells, which then die and thereby prevent the spreading of an infection. On the other hand, creation of new plasmodesmata between already separated cells that had not previously been connected, called secondary plasmodesmata, can be a means to increase cell-cell communication within a tissue when needed.

In essence, plasmodesmata are a plant specific solution to a plant specific problem: They allow the exchange of even large cellular content despite the limitations presented by the existence of a rigid cell wall and introduce another regulatory hub for phenotypic plasticity.

## *WUS* is a key player in stem cell maintenance in the SAM

Many genes have been found to play an important role in stem cell regulation in plants. Here, we would like to focus on the *WUSCHEL* (*WUS*) gene, which is one of the key regulators of stem cell fate in the *A. thaliana* shoot apical meristem with related genes executing similar functions in many other plants. We will briefly discuss its importance for stem cell maintenance and regulation, will focus on its role as a non-cell autonomous niche factor and will furthermore comment on findings regarding the mechanism of WUS protein mobility.

The *WUS* gene was identified in a forward genetic screen to find shoot meristem defective mutants in Arabidopsis using EMS mutagenesis (Laux et al. 1996). Homozygous *wus* mutants failed to maintain shoot meristems, including vegetative, inflorescence and floral meristems, all of which initiated and terminated repetitively. As a result, shoot development was delayed and proceeded in a disorganised ’stop-and-go’ manner. Organ formation itself was not affected, neither were the root meristem or root development. The gene has been named for the disorganised appearance of its mutant phenotype: compare ‘Wuschelkopf’—a german expression for a person with fuzzy or dishevelled hair. Adult *wus* mutant plants displayed large numbers of rosette leaves that clustered at the base. Likewise, inflorescences showed disorganised clusters of cauline leaves along the stem. Flowers were formed only rarely, lacked reproductive organs except one central stamen and were generally infertile (Laux et al. 1996).

**Fig. 3:**
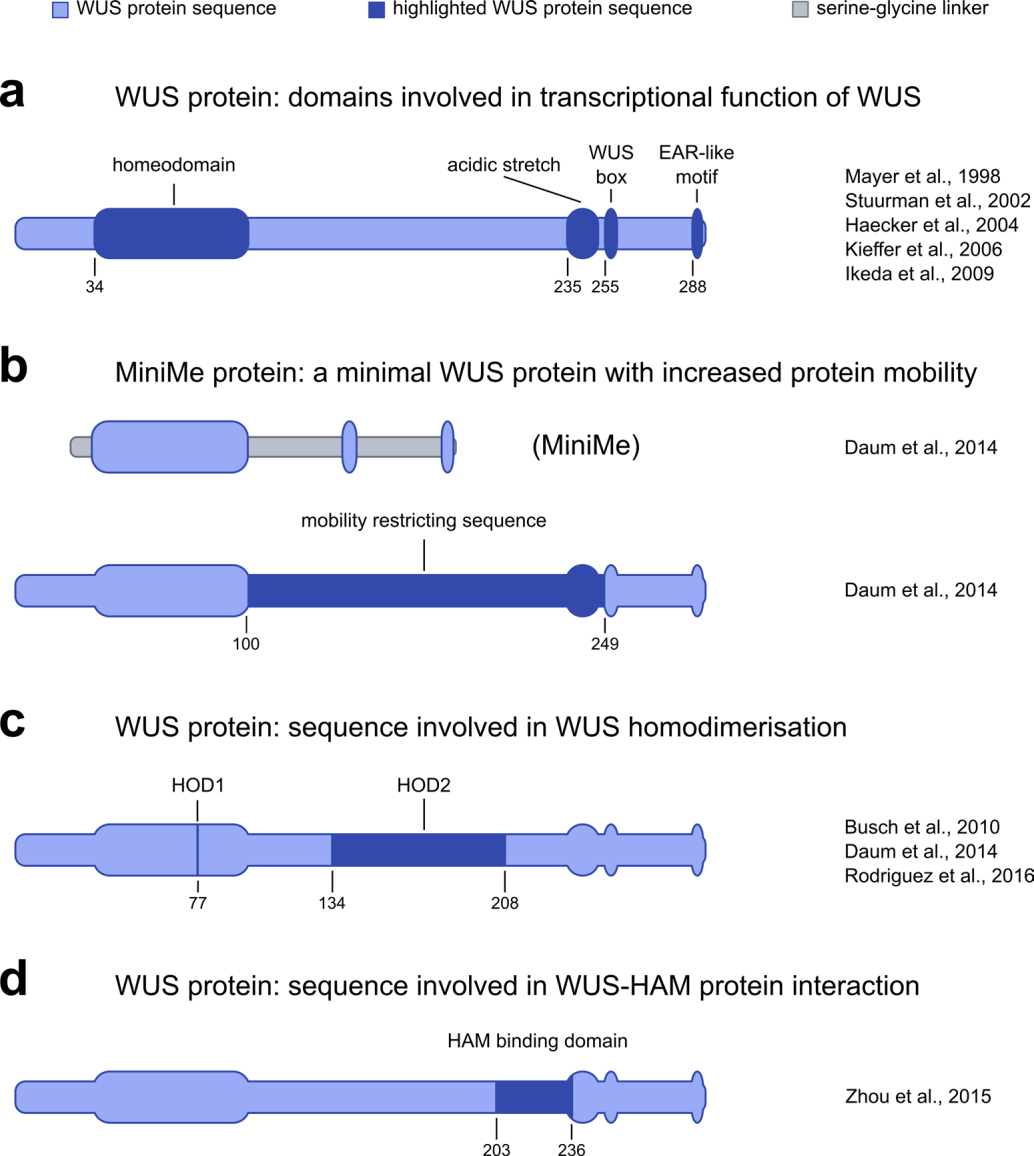
Overview of different functional domains within the WUS protein. **a** Schematic representation of domains involved in the transcriptional function of WUS. **b** Schematic representation of the highly mobile MiniMe protein—a minimal WUS protein consisting only of the WUS homeodomain, WUS box and EAR-like motif. From this, Daum and colleagues have identified a mobility restricting sequence in the WUS protein. **c** Schematic representation of WUS protein sequence involved in WUS homodimerisation. **d** Schematic representation of WUS protein sequence involved in WUS-HAM protein-protein interaction

The *WUS* gene has been localised to chromosome 2 of *A. thaliana* (Laux et al. 1996). It was identified using map-based cloning and the locus was confirmed by its ability to rescue the *wus* mutant phenotype (Mayer et al. 1998). A protein of 291 amino acids was predicted from the gene sequence and initially two distinct functional domains were described (Fig. 3a): First, a domain that showed key characteristics of a DNA binding homeodomain as well as high sequence similarity to known homeodomains. It consists of 66 amino acids (Mayer et al. 1998) rather than 60 amino acids as described for others previously (Gehring et al. 1994) and could not be phyllogenetically grouped with any other homeodomain. And second, a cluster of amino acids with acidic residues, that was predicted to form a structure generally associated with transactivation domains (Ptashne 1988). The presence of a potential DNA binding domain and a predicted transcriptional activation domain, as well as the finding that a translational fusion between WUS and a Glucuronidase (GUS) reporter gene localised to the nucleus in onion epidermis cells, led Mayer and colleagues to classify WUS as a novel homeodomain transcription factor (Mayer et al. 1998).

## *WUS* acts non-cell autonomously to regulate stem cell fate

In situ hybridisation revealed that *WUS* was expressed in the inflorescence meristem (*A. thaliana*, ecotype Landsberg) and that the RNA accumulated in a small number of cells in the centre of L3 and layers below. Even though *WUS* is essential for stem cell function, *WUS* mRNA was found exclusively in this domain but not in L1 and L2 stem cells located in the central zone above (Fig. 2a, b). Therefore, *WUS* was suggested to act non-cell autonomously and the *WUS* expression domain was named organising center (OC) accordingly (Mayer et al. 1998). This finding fit well with what was known about the concepts of stem cell regulation in the root meristem where cells of the quiescent centre keep adjacent cells from differentiating via an obviously non-cell autonomous mechanism (van den Berg et al. 1997) as well as with the general understanding of stem-cell-to-niche interactions in other organisms.

## WUS is a transcriptional activator as well as a transcriptional repressor

Phenotypic analysis of meristem mutants in other plant species has led to the identification of *WUS* orthologs, such as *TERMINATOR* (*TER*) in *Petunia* (Stuurman et al. 2002) and *ROSULATA* (*ROA*) in *Antirrhinum* (Kieffer et al. 2006). These orthologs have allowed for the identification of two more conserved motifs: A TLPLFPMH motif of unknown function, called WUS box (Haecker et al. 2004; Stuurman et al. 2002), and an EAR-like domain (ASLELTLN) (Fig. 3a) (Kieffer et al. 2006; Stuurman et al. 2002). EAR-like domains have been reported previously to act in transcriptional repression (Hiratsu et al. 2004; Ohta et al. 2001; Tiwari et al. 2004). The acidic domain (Mayer et al. 1998), the WUS box and the EAR-like domain are located at the C-terminal end of the WUS protein, which was shown to be necessary to rescue the *wus* mutant phenotype (Kieffer et al. 2006). Transient expression assays in Arabidopsis leaves confirmed the acidic domain of *WUS* to be involved in transcriptional activation and the EAR-like domain to be involved in transcriptional repression, as had been suggested before. Additionally, the WUS box was identified to have a strong repressive function (Ikeda et al. 2009). Ectopic expression of *WUS* has been reported to induce shoot stem cell identity or the formation of somatic embryos, depending on tissue identity and auxin levels (Gallois et al. 2004; Zuo et al. 2002). Interestingly, mutations in the acidic domain or in the EAR-like domain did lower, but not eliminate the ability of ectopically expressed WUS protein to cause over-proliferation, while a mutation of the WUS box completely abolished the formation of ectopic shoot tissue or somatic embryos (Ikeda et al. 2009). Instead, in some cases, plants containing ectopically expressed *WUS* with a mutated WUS box showed meristematic and flowering phenotypes similar to *wus* mutant plants. Possibly, this may be due to competition with endogenous WUS protein and a dominant negative effect of the mutated WUS box. Consequently, WUS protein expressed under the control of a 3 kb fragment of the endogenous *WUS* promoter was able to rescue the meristematic phenotype of the *wus* mutant - even though plants were still male infertile and did not produce offspring. Complementation of the mutant phenotype was not reduced when the acidic domain or the EAR-like domain were mutated, however, WUS protein with a mutation in the WUS box completely failed to rescue *wus* mutants (Ikeda et al. 2009). Therefore, it was shown that the WUS box is necessary for all biological functions of *WUS*, while the acidic domain and the EAR-like domain support transcriptional activation and repression respectively, but are not essential. Both functions of the WUS protein, transcriptional activation and transcriptional repression of target genes, have been demonstrated to be physiologically relevant (Leibfried et al. 2005; Lohmann et al. 2001) and could readily be identified *in vivo* in transcriptome analysis following *WUS* activation (Busch et al. 2010; Leibfried et al. 2005; Ma et al. 2019; Yadav et al. 2013). A closer analysis of WUS targets characterised *WUS* as a modulator of gene expression whose opposing sub-functions in transcriptional regulation were spatially separated in distinct tissues (Busch et al. 2010).

## *WUS* can act via negative feedback regulation

Curiously, the first direct WUS target was not identified in the shoot apical meristem but in floral meristems. WUS was found to activate and establish expression of the *AGAMOUS* (*AG*) gene (Lenhard et al. 2001; Lohmann et al. 2001), a regulator of floral organ identity and proliferation of floral stem cells (Bowman et al. 1989, Bowman et al. 1991; Mizukami and Ma 1995; Sieburth et al. 1995). Flowers of *ag* mutant plants failed to specify carpels and stamen, but produced sepals and petals indeterminately (Bowman et al. 1989). In contrast, *wus* mutant flowers failed to produce carpels and most stamen, but contained normal numbers of sepals and petals. The additional mutation of the *WUS* gene in *ag* mutants abolished indeterminate growth of *ag* flowers, which formed sepals and then terminated in a central petal (Laux et al. 1996). Ectopic expression of *WUS* within floral meristems led to increased organ numbers as well as organ transformation (Lenhard et al. 2001; Lohmann et al. 2001). This data suggested a strong link between *WUS* and *AG* functions in the floral meristem. Both papers showed that *WUS* establishes *AG* expression in the floral meristem and among other genes activates transcription of *AG* by direct binding to its promoter. AG in turn, once established, was found to negatively regulate WUS expression and thus prevent over-accumulation of floral stem cells, creating a temporally separated *WUS/AG* feedback loop (Lenhard et al. 2001; Lohmann et al. 2001).

Similar feedback regulation has also been described in the shoot apical meristem. Here, *WUS* was identified to act in a negative feedback loop together with the stem cell marker *CLAVATA3* (*CLV3*). CLV3 is a small peptide that is processed at the N- and C-terminus and then secreted from the stem cells in the CZ (Fig. 2a, b) to non-cell autonomously promote cell differentiation of other cells (Clark et al. 1995; Fletcher et al. 1999; Rojo et al. 2002). This spatially separated *WUS*-*CLV3* feedback loop was found to control the size of the stem cell pool in the SAM by balancing *WUS* and *CLV3* expression and with that keeping the balance between stem cell fate and differentiation (Brand et al. 2000; Schoof et al. 2000). *WUS* activity induces expression of *CLV3*, which in turn signals back to limit *WUS* expression. Therefore, high expression of *WUS* led to increased expression of *CLV3*, which then led to lower expression of *WUS*, which in turn caused lower expression of *CLV3*. In *clv3* mutants, *WUS* expression was not limited, but the expression domain increased in size, which led to enlarged meristems due to over-proliferation (Brand et al. 2000; Clark et al. 1995; Fletcher et al. 1999; Schoof et al. 2000). In *wus* mutants, expression of *CLV3* was abolished in the embryo, but was initiated later in development by another homeodomain transcription factor, *SHOOT MERISTEMLESS* (*STM*) (Brand et al. 2002). The partially redundant mechanism for *CLV3* activation (Lenhard et al. 2002) also explained the ’stop-and-go’ mode of growth of the *wus* mutant phenotype where *CLV3* positive stem cells are initiated but are not maintained and differentiate (Laux et al. 1996). At the same time, ectopic over-expression of the *WUS* gene in the SAM was found sufficient to induce the expression of *CLV3* and led to over-accumulation of stem cells reminiscent of the *clv3* mutant phenotype due to an inability to limit *WUS* levels (Schoof et al. 2000). Conversely, enhancing the expression of *CLV3* from its native promoter led to repression of *WUS* and ultimately to stem cell termination only when *CLV3* was expressed at very high levels (Müller et al. 2006).

## *WUS* is linked to hormone signalling

Apart from the regulatory interaction with *CLV3*, *WUS* activity is mediated through the regulation of plant hormone signalling. It was long known that plant hormones such as auxin and cytokinin play an important role in the regulation of cell proliferation as well as root and shoot development (Skoog et al. 1957). *WUS* was shown to directly repress the transcription of *ARABIDOPSIS RESPONSE REGULATOR*s (*ARR*s), namely *ARR7* and *ARR15*, (Leibfried et al. 2005), which are induced by cytokinin but are negative regulators of the cytokinin signalling pathway themselves (Kiba et al. 2003; To et al. 2004). At the same time, over-expression and constitutive activation of *ARR7*, mimicking a reduction in cytokinin signalling, led to seedling phenotypes comparable to the ones described for *wus* mutants (Leibfried et al. 2005). Consequently, *ARR7/ARR15* mediated cytokinin signalling was found to activate *WUS* expression (Gordon et al. 2009), via *CLV*-dependent and *CLV*-independent feedback loops, as well as the expression of *CLV3* (Zhao et al. 2010). This regulation is modulated by auxin, another phytohormone, which was identified to negatively regulate *ARR7* and *ARR15* via the transcription factor *AUXIN RESPONSE FACTOR 5/MONOPTEROS* (*MP*). Inhibition of auxin transport by *N*-1-naphthylphthalamic acid (NPA) resulted in increased *CLV3* levels and decreased expression of *WUS* (Zhao et al. 2010). Altogether, these data showed that auxin and cytokinin affect stem cell fate via *ARR7/ARR15* which in turn act on the core *CLV3*/*WUS* feedback loop. A more recent paper has described WUS as a gatekeeper of auxin function in the SAM, balancing low levels of auxin signalling required in stem cells with high levels of auxin signalling that lead to differentiation in other meristematic cells (Ma et al. 2019).

## Non-cell autonomous *WUS* function is mediated by cell-to-cell movement

One of the fundamental questions that is left unanswered by these findings relates to the non-cell autonomous activity of *WUS*: How is *WUS* able to control stem cell fate when its expression is confined to neighbouring niche cells? Or in other words: What is the nature of the *WUS* mediated mobile signal from the OC to the stem cells in the CZ?

For a number of plant transcription factors the ability for intercellular movement of the protein itself had been demonstrated already (Jackson et al. 1994; Kim et al. 2003; Lucas et al. 1995; Nakajima et al. 2001; Perbal et al. 1996; Schlereth et al. 2010; Sessions et al. 2000; Wada et al. 2002). These include the maize gene *KNOTTED1* (*KN1*) as well as Arabidopsis genes *KNAT1/BREVIPEDICELLUS* (*BP*) and *SHOOT MERISTEMLESS* (*STM*), which, like WUS, are also homeobox proteins. Therefore, Yadav and colleagues hypothesised that the WUS protein itself might be mobile. They fused a green fluorescent protein (GFP) to the N-terminus of the WUS protein and expressed it in the *wus* mutant background using a 5.6 kb promoter fragment as well as a 1.2 kb terminator fragment (Yadav et al. 2011). This construct, from now on called *GFP-WUS*, reportedly rescued the *wus* mutant phenotype, producing viable offspring. *In situ* hybridisation with a *GFP* specific probe showed an expression pattern resembling the expression pattern of the endogenous *WUS* (Mayer et al. 1998). Immunoprecipitation and antibody detection with both WUS and GFP antibodies showed no signs for a cleavage of the fusion protein and it was thus assumed that GFP signal would recapitulate the presence of WUS protein. Confocal microscopy revealed that in contrast to its mRNA, GFP-WUS protein was not restricted to the OC, but could be detected in a much broader domain, extending towards the L1 layer (Yadav et al. 2011) (compare Fig. 2c, d). Since the mRNA expression domain had not extended, this data suggested that the GFP-WUS protein itself was able to migrate from cell to cell and that this might be the mediator of *WUS* non-cell autonomous function. Similar to what had been shown for the *SHORT-ROOT* (*SHR*) transcription factor in Arabidopsis root tissue (Gallagher et al. 2004), addition of an N-terminal nuclear localisation signal (NLS) inhibited GFP-WUS protein movement in the SAM (Yadav et al. 2011). NLS-GFP-WUS fusion protein was not able to fully rescue the *wus* mutant phenotype, however, in the rare case that transgenic plants produced a small number of flowers, these contained the full set of organs (Yadav et al. 2011) in contrast to what has been reported for the original *wus* mutant (Mayer et al. 1998). This seemed to suggest that the NLS-GFP-WUS protein was functional, but failed to rescue the *wus* phenotype due to its decreased ability for cellular migration. Similarly, a fusion protein that contained an additional copy of GFP (2xGFP-WUS), resulting in a protein with a higher molecular weight, was impaired in movement and in its ability to rescue *wus* mutants (Yadav et al. 2011), giving weight to the idea that WUS might move through plasmodesmata, which are known to restrict passage via size exclusion.

## Intercellular movement of WUS protein occurs via plasmodesmata and is essential for *WUS* function

The possibility that WUS proteins moves from cell to cell through plasmodesmata was investigated more thoroughly by Daum and colleagues: By blocking plasmodesmata specifically in the OC, expressing constitutively active *CALLOSE SYNTHASE 3* (*cals3m*) (Vatén et al. 2011) from the *WUS* promoter, they obtained plants showing *wus* mutant phenotypes despite the presence of the endogenous *WUS* gene (Daum et al. 2014). Furthermore, in experiments using an inducible version of *cals3m* (*icals3m*) driven only in the *CLV3* positive stem cells, first signs of cell differentiation could be observed on the microscopic level as early as 3 days after induction and stem cells were lost after 5 days. After 15–18 days, depletion of all dividing cells had led to shoot meristem termination and therefore arrested development. Interestingly, side shoots or axillary shoots initiated after the transient induction as well as non-induced plants grew and developed completely normal, clearly underlying the importance of cellular connectivity (via plasmodesmata) in the SAM for stem cell maintenance (Daum et al. 2014).

To be able to directly asses WUS protein distribution in meristems with blocked plasmodesmata, Daum and colleagues created their own *WUS* rescue line using a 4.4 kb fragment of the *WUS* promoter and a 2.8 kb fragment of the *WUS* terminator. To avoid potential interference of the protein tag with WUS protein domains and especially with the N-terminal homeodomain, they added the GFP tag at the C-terminus of WUS and separated both proteins by a 30 amino acid linker. This fusion protein, called WUS-linker-GFP, rescued the *wus* phenotype and in situ hybridisation using *WUS* mRNA probes confirmed its expression in the OC. Additionally, Daum and colleagues were able to perform immunohistochemical analysis of the distribution of the endogenous WUS protein in wild type plants, for the first time showing native WUS in stem cells. This result strongly supported cell-to-cell movement of the endogenous protein into L1 and L2 and verified that their C-terminal rescue (WUS-linker-GFP) recapitulated the distribution of endogenous WUS (Daum et al. 2014) as well as the distribution of the previously published N-terminal GFP-WUS fusion protein (Yadav et al. 2011). When Daum and colleagues used the *WUS-linker-GFP* rescue line to drive *icals3m* in *CLV3* cells, blocking plasmodesmata in the stem cells, they found that as early as 8 hours after induction, WUS-linker-GFP protein could no longer be detected in the L1 and L2, but was restricted to its expression domain in the OC. Induced plants developed terminated meristems as described before and interestingly *WUS-linker-GFP* rescue plants arrested earlier than wild type plants, most likely due to the increased molecular weight in comparison to endogenous WUS (Daum et al. 2014) and similar to what had been observed for 2xGFP-WUS (Yadav et al. 2011).

It had been long known that *WUS* function is essential for stem cell maintenance in the SAM (Laux et al. 1996; Mayer et al. 1998; Schoof et al. 2000) and it seemed increasingly clear that cell-to-cell mobility of the WUS protein is necessary for its function (Daum et al. 2014; Mayer et al. 1998; Yadav et al. 2011). However, it was not trivial to clearly distinguish between effects caused by changes in protein functionality and effects caused by disturbed protein mobility. Therefore, Daum and colleagues designed an experiment to specifically degrade a WUS fusion protein in *CLV3* expressing stem cells only, using the TIPI-Degron approach (Taxis et al. 2009; Taxis and Knop 2012). For this, WUS was tagged at the N-terminus with a yellow fluorescent protein (YFP), a TEV protease recognition site and an N-degron sequence. This fusion protein allowed for stem cell specific degradation of WUS, since after TEV protease cleavage, the N-degron would promote rapid proteasome mediated degradation of WUS. Daum and colleagues reported that in the absence of a TEV protease or if the TEV recognition site was missing, the fusion protein showed nuclear localisation, moved from its site of expression in the OC to the stem cells in the central zone and was able to rescue the *wus* phenotype in a large fraction of T1 plants. In the presence of TEV protease in stem cells, fluorescent signal was still detected in L1 and L2 but was no longer nuclear, indicating the presence of cleaved free YFP rather than the original fusion protein. A significant fraction of such plants, compared to controls, suffered meristem termination, indicating successful degradation of WUS protein after cleavage. This clearly showed that *WUS* function is required in stem cell, which the protein can only reach by cell-to-cell movement and that therefore unperturbed protein mobility is absolutely essential. Furthermore, the fact that not all plants terminated, seemed to suggest a certain dosage dependency for WUS function in stem cells (Daum et al. 2014). Using the deGradFP technology (Caussinus et al. 2012) instead of TIPI-Degron, a recent study obtained the same results (Ma et al. 2019). Taken together, the evidence based on increasing the size of WUS, closing plasmodesmata in stem cells, or removing active WUS protein from these cells by two independent methods all point to the fact that WUS protein is required in stem cells and moves there from its site of expression in the OC via plasmodesmata.

## WUS mobility is regulated by specific amino acid sequences within the protein

It was unclear whether the ability to move from the OC to the CZ is an inherent property of the WUS protein or of the meristem itself, especially since STM movement within the shoot apical meristem had already been reported (Kim et al. 2003). Comparison of different fusion proteins showed that the ability to move from the *WUS* expression domain towards the L1 correlated with sequence similarity to *WUS* (Daum et al. 2014). The closely related WUS RELATED HOMEOBOX 5 (WOX5) protein was able to move towards the L1 and even partially rescued the *wus* mutant phenotype. In contrast, WOX13 which is a more distant member of the WUS/WOX protein family, lacking the WUS box, could barely be detected in the L1 and completely unrelated proteins such as for instance the basic helix-loop-helix transcription factor HECATE 1 (HEC1) or a 2xGFP-NLS fusion were never found outside of the OC when expressed from the *WUS* promoter (Daum et al. 2014). Interestingly, WUS protein that had been inactivated by mutating the WUS box (Ikeda et al. 2009) (WUS∆box) showed a similar movement behaviour to the native protein, allowing miss-expression experiments without ectopic induction of stem cell fate. Therefore, Daum and colleagues tested the movement abilities of WUS∆box, WOX13 and 2xGFP-NLS again, but expressed from the epidermal *MERISTEM LAYER 1* (*ML1*) (Sessions et al. 1999) promoter and found that WUS∆box moved further than WOX13 while 2xGFP-NLS did not move at all. They concluded that cell-to-cell mobility is a property specific to the WUS protein, encoded in its sequence and not dependent on tissue specific properties of the SAM (Daum et al. 2014).

Even though a number of plant proteins, including transcription factors, have been shown to be mobile, no consensus sequence responsible for mobility has been identified so far (Xu et al. 2010) and yet WUS mobility seemed to be encoded in its sequence. To identify these movement promoting sequence(s) Daum and colleagues systematically mutated or replaced domains within the WUS protein by serine-glycine linker. Mutations of the WUS box (WUS∆box) or of the EAR-like domain (WUS∆EAR) did not qualitatively change protein distribution when expressed from the *WUS* promoter. Deletion of the homeodomain (hd) did drastically increase protein distribution in the SAM, however, the chimeric protein had completely lost nuclear localisation so that movement by passive diffusion could not be excluded. Conversely, when the WUS homeodomain was fused to 2xGFP-NLS, by itself immobile, movement of the fusion protein was observed, while the same experiment with the WOX13 homeodomain failed to increase mobility. Surprisingly, the fusion protein WUShd-2xGFP-NLS moved further than WUS-GFP-NLS, even though the protein was larger. This suggested, that not only did the WUS homeodomain act as a movement promoting sequence, but that additional movement restricting sequences were present in the WUS protein (Daum et al. 2014). Since WUS box and EAR-like domain had already been shown to have only little effect on mobility, restriction of movement had to be encoded elsewhere: When Daum and colleagues replaced all WUS sequence except the homeodomain, the WUS box and the EAR-like domain by non-specific linker sequence, the resulting protein, which they called MiniMe (Fig. 3b), was not only detected in the whole SAM, but led to massive stem cell over-proliferation in a large fraction of plants and ectopic activation of the *WUS* promoter in a salt-and-pepper pattern. In contrast, a MiniMe version carrying the ∆box mutation was present in the whole meristem as well, but the SAM did not show any signs of over-proliferation as well as no expansion of the *WUS* expression domain, clearly attributing the enlarged domain of protein occurrence to increased mobility. Additionally, a MiniMe-GFP-NLS fusion protein was distributed throughout the SAM whereas the WUS-GFP-NLS fusion described above could hardly be detected outside of the OC. Taken together, these data confirmed the presence of potent movement inhibitory sequences outside of the WUS homeodomain, WUS box and EAR-like domain which upon deletion results in massively increased mobility. This sequence was further narrowed down to amino acids 100-249 between the WUS homeodomain and the WUS box (Daum et al. 2014) (Fig. 3b).

## WUS protein mobility may be influenced by the formation of homo- and heterodimers

Since the mobility restricting sequence identified overlapped with a sequence that had been shown to be involved in homodimerisation of Arabidopsis and rice WUS proteins (amino acid 117-292) using yeast two-hybrid (Y2H) studies (Busch et al. 2010; Nagasaki et al. 2005), Daum and colleagues speculated that protein dimerisation might be a potential mechanism to restrict WUS mobility. They confirmed homodimerisation of Arabidopsis WUS protein in vivo and observed a reduction in dimer formation when analysing interaction of WUS and MiniMe. This however could be restored by reintroducing the 149 amino acid long sequence stretch identified using the MiniMe protein previously, suggesting that homodimerisation of WUS protein might indeed contribute to the regulation of its own mobility (Daum et al. 2014). Another study aimed to investigate potential effects of WUS homodimerisation more closely (Rodriguez et al. 2016). Using Y2H analysis they identified two sequence stretches important for homodimerisation (Fig. 3c) one of which was the WUS homeodomain, named homodimerisation domain 1 (HOD1), while the other (HOD2) narrowed down a previously published homodimerisation domain (Busch et al. 2010). Rodriguez and colleagues could show that homodimerisation via the homeodomain (HOD1) can be abolished by introducing a single point mutation, recreating the weak *wus* mutant allele *wus-7* (Graf et al. 2010). Deletion or mutation of either HOD1 or HOD2 led to reduction in dimer formation (Daum et al. 2014; Perales et al. 2016; Rodriguez et al. 2016), which however in in vitro studies seemed to be concentration dependent (Perales et al. 2016). Rodriguez and colleagues observed that *WUS* with a mutation in HOD2 failed to fully rescue *wus* mutants, similar to what had been reported for HOD1 (*wus-7*) and plants carrying mutations in both, HOD1 and HOD2, did not rescue at all (Rodriguez et al. 2016), underlining the physiological importance of WUS homodimerisation. Additional analysis of fluorescently labelled WUS carrying HOD mutations expressed from the *WUS* promoter led Rodriguez and colleagues to suggest that DNA binding and homodimerisation are important for nuclear accumulation of WUS protein as well as limiting its spatial distribution in the SAM (Rodriguez et al. 2016). These conclusions are in line with a previous report documenting the loss of nuclear localisation upon deletion of the homeodomain (Daum et al. 2014) and support the idea that WUS homodimerisation restricts protein mobility as originally speculated by Daum and colleagues.

However, while both studies in some aspects complement each other and come to similar conclusions, there are striking differences as well: For instance, when Daum and colleagues removed a large sequence stretch including the HOD2 from WUS, the resulting fusion protein (MiniMe) was found throughout the SAM and led to massive over-proliferation. In contrast, the HOD2 deletion created by Rodriguez and colleagues in a later study was reported to recapitulate wild type WUS protein distribution and resulted in only partial rescue of the *wus* phenotype rather than over-proliferation. In light of these contradictory results, Rodriguez and colleagues suggested that the MiniMe protein might be artificially stabilised due to its nature as a C-terminal fusion and the masking of a potential protein destabilisation domain located at the C-terminus (Rodriguez et al. 2016) and indeed, unexpected behaviour of any protein fusion is hard to rule out in general. Another conceivable explanation however could be found in the deleted sequence itself: The MiniMe protein lacks WUS amino acids 100-249, while HOD2 has been reported to encompass amino acids 134-208, meaning that additional sequence stretches which may facilitate different protein-protein interactions are missing in the MiniMe protein. A likely candidate for such interaction presents itself in form of the HAIRY MERISTEM 1 (HAM1) protein, which has been reported to bind to WUS at amino acids 203-236 (Fig. 3d) (Zhou et al. 2015). Interestingly, HAM1 and HAM2 were observed broadly expressed in lower layers of the SAM, overlapping with WUS expression, but in contrast to WUS, HAM protein did not move beyond its expression domain, creating distinct zones where only HAM1/2, only WUS or both proteins can be found (Zhou et al. 2015; 2018). Considering the data on WUS homodimerization, it is conceivable that WUS and HAM proteins form protein complexes of variable size and composition to regulate the distribution of WUS protein within the meristem and with that stem cell maintenance. The differences observed in protein behaviour of the different deletion constructs may stem from the differential ability to form such complexes due to a lack of specific binding sites, differences in size or differences in protein folding.

## Are the domains responsible for the transcriptional activity of WUS involved in its mobility?

Further experiments by Rodriguez and colleagues led to additional insights: Free GFP as well as NLS-GFP expressed from the *WUS* promoter were both found uniformly distributed in the SAM. A WUS truncation consisting of the first 134 amino acids, including the homeodomain containing HOD1, was broadly distributed in the meristem and seemed to spread almost as uniformly as NLS-GFP. From this the authors concluded that the missing C-terminal amino acids contained information for spatial patterning of the protein. In light of their findings with regards to dimer formation and its implicated role in mobility, it would have been interesting to discuss the possibility of heterodimer formation with the endogenous WUS protein present in these plants. If the formation of such heterodimers did occur, it could be speculated why the distribution of this truncation did not resemble the distribution of endogenous WUS protein more closely. Interestingly however, when Rodriguez and colleagues deleted everything but the last 63 amino acids at the WUS C-terminus, which contain all domains necessary for transcriptional activity of the protein (Fig. 3a) (Ikeda et al. 2009; Kiefer et al. 2006) the resulting fusion protein was found in a domain very similar to the distribution of endogenous WUS. Therefore, the authors argue that the last 63 amino acids were sufficient to maintain spatial distribution of the WUS protein (Rodriguez et al. 2016). That is surprising since this C-terminal stretch has so far only been associated with transcriptional activity (Ikeda et al. 2009; Kiefer et al. 2006) and the fusion protein analysed lacked all sequence stretches that had been associated with WUS protein mobility in an earlier study (Daum et al. 2014), but also in the same study (Rodriguez et al. 2016). It may be that in the absence of other factors influencing protein mobility, WUS protein distribution is predominantly controlled by the interaction with co-repressors or co-activators. In any case, the data presented stresses that defining protein domains and comparing protein distribution is no trivial feat. It seems of utmost importance that we rely on imaging data of highest quality and develop robust methods for *in vivo* quantification of proteins.

Since they suspected the C-terminal stretch of WUS to be responsible for protein distribution, Rodriguez and colleagues next created individual deletions of the acidic domain, the WUS box and the EAR-like domain. In three single images shown, all three constructs were barely detectable above what has to be considered background intensity and only deletion constructs of the WUS box and the acidic domain showed faint nuclear signal in the OC. From this sparse evidence, the authors concluded that the fusion proteins must have been destabilised and claimed that deleting any of the C-terminal domains had resulted in the exposure of a hypothesised destabilisation signature (Rodriguez et al. 2016).

In light of their results, Rodriguez and colleagues set out to repeat an experiment already performed in a previous paper and analysed mutations in the WUS box and the EAR-like domain—which will be called *WUS∆box* and *WUS∆EAR* respectively for reasons of simplicity - rather than complete deletions. In their paper they write that *GFP-WUS∆box* expressed from the *WUS* promoter exhibited „dramatic nonnuclear accumulation”, that GFP-WUS∆EAR protein accumulated at higher levels in the nucleus compared to GFP-WUS with plants in very rare occasions showing enlarged meristems and that GFP-WUS∆box∆EAR behaved indistinguishable from the fusion protein carrying only the ∆box mutation (Rodriguez et al. 2016). Careful inspection of the images presented in the study, confirm that nuclear localisation is lost in GFP-WUS∆box and only weak cytoplasmic signal can be observed. GFP-WUS∆EAR and GFP-WUS∆box∆EAR on the other hand are nuclear and seem to spread in a broader domain, however without additional information about image acquisition, without normalisation between images and without signal quantification, any speculation about differences in intensity and therefore differences in absolute and sub-cellular protein levels seem premature. In any case, Rodriguez and colleagues conclude, that the WUS box was responsible for nuclear retention and EAR-like domain facilitated nuclear export and that the function of both domains together balanced nuclear versus cytoplasmic WUS levels.

These interpretations, but also the imaging data shown, are not in agreement with data presented in an earlier study, where it was shown that the same mutations in the WUS box and the EAR-like domain did not significantly change WUS protein distribution (Daum et al. 2014). Rodriguez and colleagues expressed their concern, that the C-terminal nature of the WUS-linker-GFP constructs used by Daum and colleagues, in contrast to their own N-terminal GFP-WUS constructs, might have interfered with the proposed activity of the C-terminal domains. With their concern they raise a valid point that is true for all fusion proteins, but is unfortunately often ignored: When tagging a protein at the N-terminus or at the C-terminus, subsequent analysis needs to ensure unchanged and proper protein function. This is usually done in rescue experiments and indeed, both groups report that their respective fusion protein rescues the *wus* mutant phenotype. In their paper, Daum and colleagues argue to have chosen the C-terminal fusion including a flexible linker, to not interfere with the important N-terminal homeodomain of the WUS protein. Again, a systematic approach in comparing different variants of WUS fusion protein using high quality imaging and quantification of protein distribution seems required to draw solid conclusions.

## Joining the club: What can we learn from other mobile plant transcription factors?

Proteins that can travel from one cell to another have been observed commonly in the plant kingdom. Indeed, when a study analysed 61 transcription factors whose expression was enriched in root tissue, they found that in 25% of the cases the distribution of protein did not match the RNA expression domain (Lee et a. 2006). Of course, the ability of a protein to move to another cell does not necessarily imply any physiological role, however, we know of a number of transcription factors that rely on protein movement to fulfil their function.

Prominent examples include the homeodomain transcription factor KNOTTED1 (KN1) which was observed to move from L2 to L1 in the shoot apical meristem in *Zea mays* (Jackson et al. 1994; Lucas et al. 1995). KN1 movement occurred through plasmodesmata, required a conformational change (Kragler et al. 1998) and the ability for cell-to-cell movement in the SAM was conserved among Arabidopsis KNOTTED 1-like homeobox (KNOX) proteins (KNAT1/BREVIPEDICELLUS (BP) and SHOOT MERISTEMLESS (STM) (Kim et al. 2003). The KN1 homeodomain was found to be both necessary and sufficient for trafficking of the protein as well as its mRNA (Kim et al. 2005). A later study identified the CCT8 subunit of the type II chaperonin complex as a physical interactor of KN1 as well as STM and CCT8 was shown to be essential for their trafficking, since protein movement was abolished in weak *cct8* mutants (Xu et al. 2011). Xu and colleagues proposed that KN1 is targeted to plasmodesmata, (partially) unfolded for its passage and refolded again on the other side. Since like for STM, the homeodomain was shown to be required for movement of WUS (even though STM and WUS homeodomains share little homology), Daum and colleagues analysed the mobility of their WUS-linker-GFP fusion protein in the background of a *cct8 wus* double mutant. Interestingly, the distribution of WUS-linker-GFP was unchanged in the *cct8* mutant, revealing that mobility of WUS and of KN1 as well as STM are likely to be mediated by independent mechanisms (Daum et al. 2014).

Similarly, the *cct8* mutation did not change the mobility of SHR (Xu et al. 2011), a GRAS family transcription factor that was shown to move within the root of *A. thaliana* from stele cells to the epidermis, initial cells and cells of the quiescent centre (Gallagher et al. 2004; Nakajima et al. 2001). SHR movement to these regions was found to be necessary for normal development and patterning of the Arabidopsis root (Gallagher et al. 2009; Nakajima et al. 2001) and required the GRAS domain, another conserved DNA binding domain (Gallagher et al. 2009). Nuclear localisation of SHR was shown to be necessary to enable cell-to-cell mobility, but not sufficient (Gallagher et al. 2004). On the other hand, Gallagher and colleagues also reported that nuclear trapping of SHR can prevent its movement and indeed the protein-protein interaction with the nuclear protein SCARECROW (SCR) was suggested as a mechanism to limit excessive spreading of SHR protein (Cui et al. 2007; Gallagher et al. 2004). Therefore, it is thought that SHR mobility is controlled by nuclear-cytoplasmic partitioning (Gallagher et al. 2009), similar to what Rodriguez and colleagues have later proposed for WUS movement (Rodriguez et al. 2016). Subsequent studies revealed that SRH movement is mediated by the endosome associated SHORT-ROOT INTERACTING EMBRYONIC LETHAL (SIEL), which also interacts with several other root specific transcription factors such as CAPRICE (CPC) and TARGET OF MONOPTEROUS 7 (TMO7) (Koizumi et al. 2011). Additionally, nuclear localisation had been found to be crucial for both proteins (Kurata et al. 2005; Lu et al. 2018) and the addition of a strong NLS, but also of a nuclear export signal (NES) could disturb TMO7 mobility. Furthermore, TMO7 movement occurred unidirectionally towards the root tip and, similar to WUS, in a sequence specific manner, despite the small size of the protein (~ 11 kDa) that would certainly allow passive diffusion through plasmodesmata (Lu et al. 2018).

## Outlook

Since the initial description of the symplastic movement of KN1, many other plant transcription factors have been shown to act non-cell autonomously via protein movement and it seems likely that the number of such factors will further increase in the future. So far, mobile transcription factors described share striking conceptional similarities, but also great differences and it remains to be seen how many diverse mechanisms to facilitate cell-to-cell mobility of proteins evolution has invented.

Similarly, it becomes clear that many of the molecular mechanisms underlying stem cell homeostasis in plants are still unresolved, despite more than 25 years of work by many labs. Even when only focusing on the role of WUS, arguably one of the best studied regulators, central questions remain to be answered: How is protein mobility regulated and does this represent a significant node in stem cell regulation? How is WUS able to switch from transcriptional activator to repressor and where and when does this transformation take place? What are the target genes and pathways relevant for the diverse sub-functions of WUS in the SAM, in flowers, ovules and stamens? With powerful new technologies, such as cell type specific genome editing and single cell analyses of RNA and DNA, becoming available we may be able to address some of these questions and move on to study how the activity of plant stem cells is orchestrated in dynamic natural growth conditions.
